# Updated analysis of pediatric clinical studies registered in ClinicalTrials.gov, 2008–2019

**DOI:** 10.1186/s12887-021-02658-4

**Published:** 2021-04-30

**Authors:** Yang Zhong, Xingyu Zhang, Lijun Zhou, Lei Li, Tao Zhang

**Affiliations:** 1 Department of Epidemiology and Health Statistics, West China School of Public Health and West China fourth Hospital, Sichuan University, Chengdu, Sichuan Province, China; 2 Department of Systems, Populations and Leadership, University of Michigan School of Nursing, Ann Arbor, MI USA; 3 Sichuan Center for Disease Control and Prevention, Chengdu, Sichuan Province, China; 4 Department of Anesthesiology, Chengdu Women’s and Children’s Central Hospital,School of Medicine, University of Electronic Science and Technology of China, Chengdu, Sichuan Province, China

**Keywords:** ClinicalTrials.gov, Pediatrics, Clinical trials, Trial registries

## Abstract

**Background:**

Since the national clinical trials registry (ClinicalTrials.gov) launched in February 2000, more than 360,000 research studies in the United States and over 200 countries have registered. As the characteristics of pediatric clinical studies keep changing over time and the results-reporting mechanism is under evolving, to know about the relevant updates of data elements and the effect of policies on the quality of reporting results is significant.

**Methods:**

In this research, 53,060 clinical studies related to children registered from January 2008 to December 2019 were downloaded from ClinicalTrials.gov on August 1st, 2020. Different types of studies and critical categorical variables were identified, based on which, Cochran-Armitage test was performed to explore temporal trend of study characteristics and common pediatric clinical conditions in four time subsets. Further, to examine heterogeneity among subgroups (funding sources, funding sites, pediatric clinical conditions,etc), chi-squared test was applied.

**Results:**

A total of 36,136 clinical trials and 16,692 observational studies were identified during the study period. The pediatric clinical trials increased from 7,029 (January 2008–December 2010) to 11,738 (January 2017–December 2019). The number of missing data has declined, with the maximum extent decline from 3.7 to 0.0% (Z = − 15.90, *p* <  0.001). Drug trials decreased from 48.8 to 28.9% (Z = − 24.68, *p* <  0.001). Behavioral trials, on the other hand, increased from 12.6 to 20.4% (*Z* = 12.28, *p* <  0.001). Most pediatric clinical trials were small-scale (58.9% enrolling 1–100 participants), single-site (61.4%) and funded neither by industry nor by the NIH (59.2%). The proportion of reporting study results varied by study type (*χ*^2^ = 1,256.8, *p* <  0.001), lead sponsor (*χ*^2^ = 4,545.6, *p* <  0.001), enrollment (*χ*^2^ = 29.4, *p* <  0.001) and trial phase (*χ*^2^ = 218.8, *p* <  0.001).

**Conclusion:**

Pediatric clinical studies registered in ClinicalTrials.gov were dominated by small-scale interventional trials, containing significant heterogeneity in funding sources, funding sites, pediatric clinical conditions and study characteristics. Although the results database has evolved in the past decade, efforts to strengthen the practice of systematic reporting must be continued.

**Supplementary Information:**

The online version contains supplementary material available at 10.1186/s12887-021-02658-4.

## Background

Clinical trials are considered as the gold standard for assessing the safety and effectiveness of new therapies as well as generating evidence-based knowledge in medicine [1]. However, because of the homogeneity of pediatric population, safety concerns, strict ethical requirements and lack of commercial interest, most of the adult researches cannot be directly generalized to the pediatric population [2–5]. Therefore, to make customized intervention decisions on child patients, people’s immediate and close attention is required on pediatric clinical studies.

The establishment of the ClinicalTrials.gov back in 2000 provided significant insights into the pediatric clinical trials. Meanwhile, the policy required sponsors or trial designers to register trials and record key data elements (effective September 27, 2007), basic results (September 27, 2008) and adverse events (September 27, 2009) [6–8].

Many previous studies have examined fundamental characteristics of interventional clinical trials registered in ClinicalTrials.gov or profiled registered clinical trials for specific diseases [9–11]. From January 2008 to December 2019, a sum of 53,060 clinical studies related to children have registered in ClinicalTrials.gov. In 2012, a study by Pasquali et al. found that an assessment of the clinical trial site data set could describe the overall mix of clinical trials related to children in the United States, which was previously impossible [12]. Afterwards, descriptive analyses of clinical registration trial characteristics for different pediatric diseases began to emerge [13–15]. The development of pediatric clinical studies and regulations on registration of ClinicalTrials.gov had offered the opportunity to characterize the landscape of pediatric studies. And an updated comprehensive analysis of the recent decade registry would help stakeholders gain insight into the outline of pediatric studies about registration, critical changing over time and heterogeneity among subgroups. Based on this, more objective decisions could be made to further the implementation of pediatric studies.

According to available data sets and the purpose of updating analysis, in this article, we updated and summarized the pediatric clinical studies in two approaches. First of all, we examined fundamental characteristics (data elements reported in study registration) of pediatric clinical studies, including subgroups associated with temporal trend, funding sources, funding sites and common pediatric conditions. Then, according to the laws, regulations, and policies related to submit study results (eTable 1), we briefly analyzed the proportion of reporting study results. The two steps contributed to extract reliable evidence from updated pediatric clinical studies which may be useful to stakeholders, including policy makers, industry, and investigators, in informing future decisions regarding the conduct of studies in children.

## Materials and Methods

### Data collection

Methods used by ClinicalTrials.gov to register clinical studies have been described previously [16, 17]. Briefly, sponsors and investigators from around the world entered data through a web-based data entry system. Based on the data source, an XML data set comprising 53,060 studies, registered between January 1, 2008 and December 31, 2019, was downloaded from https://clinicaltrials.gov/ on August 1st, 2020. To identify pediatric clinical studies, we used the ‘Age Group:birth-17’ criteria. In the retrieval studies, the *Age Group* field included three types: (Child), (Child, Adult) and (Child, Adult, Older Adult).

### Data arrangement

① All studies categories

We applied the *Study type* field, consisting of four categories: interventional, observational, expanded access, and not available (NA), from the ClinicalTrials.gov registry to identify interventional trials and observational studies (See eTable 2 for detailed categorization criteria).

② Critical categorical variables for interventional trials

The critical categorical variables for interventional trials in this article were funding sources (industry, NIH [National Institutes of Health] and other) and common pediatric clinical conditions (infectious, cancer, immune and respiratory). In this case, the critical categorical variables were specified in two aspects.

For one thing, studies were categorized by Lead sponsor as well as funding sources according to ClinicalTrials.gov and the NLM (National Library of Medicine). For ClinicalTrials.gov, it stored funding organization information in two data elements (Lead sponsor and Collaborator). Based on the classification of submitted agency names by the NLM, the *Lead sponsor* field consisted of four categories including industry, NIH, US federal (excluding NIH) and other. Further, to derive probable funding sources from the data elements (Lead sponsor and Collaborator), we used the following algorithm [10]: if the lead sponsor was from industry, or the NIH was neither a lead sponsor nor collaborator and at least one collaborator was from industry, then the study was categorized as industry funded. If the lead sponsor was not from industry, and NIH was either a lead sponsor or a collaborator, then the study was categorized as NIH funded. Otherwise, if the data elements (Lead sponsor and Collaborator) were non-missing, then the study was considered to be funded by other. Therefore, the funding sources consisted of three categories (industry, NIH and other).

For another, studies were categorized according to submitted *Condition* field. To select the common pediatric clinical conditions, frequency statistics were carried out on *Condition* field of the retrieval studies. While the top six common conditions were selected to explore temporal trend in four time subsets. It must be noted that percentages of all conditions were not sum to 100% as categories were not mutually exclusive, giving rise to a reclassification of the studies into two categories (yes or no) to focus on a specific condition. For example, after setting *Study type*, *Age group* and *First posted* fields, we retrieved ‘infectious’ term in *Condition* field. If the study appeared in the previous retrieval studies (NCT number for inclusion and exclusion), it would be ‘yes’ subcategory of infectious trials, otherwise it would be ‘no’. Additionally, to examine heterogeneity among pediatric clinical conditions, the top four common conditions in interventional trials were focused.

③ Definition of completed studies

To examine the proportion of reporting study results, we identified completed studies by the *Primary completion date* field (or, where this was not recorded, the *Completion date* field). The primary completion date, as defined by ClinicalTrials.gov, was the date of collection of primary outcome data on the last patient to be enrolled. The completion date was the date of completion of the study, as defined by the individual trial’s investigators. Further, we pushed back the completion date by one year to more visually observe the results reported one year after the primary completion date (Fig. 2), as both NIH regulations and trial reporting policies required sponsors or researchers to submit results data within one year of the primary completion date. Besides, according to FDAAA (Food and Drug Administration Amendments Act) 801 in 2007, some trials covered by the FDAAA were required to report results within one year (‘trials subject to mandatory reporting’). To further explore the provenance of those studies with reported results, we identified trials as falling within the mandatory reporting rules if they were covered by the FDAAA (trials of a drug, device, or biological agent and were of phase 2 or later).

④ Missing data

Within these data sets, a few data elements were missing because of limitations in the data set or logistical problems in obtaining analyzable information. In this study, missing data of those data elements were defined as ‘missing’ subcategories to explore their temporal trend.

### Data analysis

All data sets were downloaded in ‘All Available Columns’ and ‘Tab-separated values’ formats and analyzed in R software, version 3.4.0. As a basis, we reported description of fundamental characteristics. Further, we used the Cochran-Armitage test to examine temporal trend for proportion of study characteristics and common pediatric clinical conditions in four time subsets. At the same time, the chi-squared test was applied to examine heterogeneity in proportion of funding sources (industry, NIH and other), funding sites (single-site and multisite), pediatric clinical conditions (infectious, cancer, immune and respiratory) and study characteristics (study type, lead sponsor, enrollment and phase).

## Results

### Characteristics for all studies

From January 2008 to December 2019, among 53,060 studies, there were 36,136 (68.1%) interventional trials, 16,692 (31.5%) observational studies and 232 (0.4%) expanded accesses. The amount of registered studies increased from 10,145 to 17,894 during the whole study period. Basic characteristics of interventional trials (*n* = 36,136) and observational studies (*n* = 16,692) registered in ClinicalTrials.gov during the period divided by four time subsets (January 2008–December 2010, January 2011–December 2013, January 2014–December 2016, January 2017–December 2019) were presented in Table 1 and eTable 3–4. We examined temporal trend by *Study design* (including *Primary purpose*, *Allocation* and *Masking*, etc), *Intervention*, *Enrollment* and *Lead sponsor*. The distribution of these variables has changed over time.

**Table 1:** Characteristics of interventional trials registered in ClinicalTrials.gov, 2008–2019

		Trials, No.(%)					***Z*** value ^**a**^	***P*** value
		All 2008–2019 (***n =*** 36,136)	2008–2010 (***n*** = 7,029)	2011–2013 (***n*** = 7,486)	2014–2016 (***n*** = 9,883)	2017–2019 (***n*** = 11,738)		
**Primary purpose**								
Treatment	Yes	21,126(60.0)	4,608(68.1)	4,476(62.7)	5,614(58.7)	6,428(54.8)	−16.12	< 0.001
	no	14,074(40.0)	2,160(31.9)	2,658(37.3)	3,951(41.3)	5,305(45.2)		
Prevention	Yes	6,110(17.4)	1,227(18.1)	1,376(19.3)	1,514(15.8)	1,962(16.7)	−3.57	< 0.001
	no	29,090(82.6)	5,541(81.9)	5,758(80.7)	805(84.2)	9,771(83.3)		
Missing	Yes	936(2.6)	261(3.7)	352(4.7)	318(3.2)	5(0.0)	−15.90	< 0.001
	no	35,200(97.4)	6,768(96.3)	7,134(95.3)	9,565(96.8)	11,733(100.0)		
**Intervention**								
Drug	Yes	13,348(36.9)	3,427(48.8)	3,066(41.0)	3,457(35.0)	3,398(28.9)	−24.68	< 0.001
	no	22,788(63.1)	3,602(51.2)	4,420(59.0)	6,426(65.0)	8,340(71.1)		
Behavioral	Yes	6,194(17.1)	889(12.6)	1,171(15.6)	1,739(17.6)	2,395(20.4)	12.28	< 0.001
	no	29,942(82.9)	6,140(87.4)	6,315(84.4)	8,144(82.4)	9,343(79.6)		
**Enrollment**								
1–100	Yes	20,676(58.9)	3,820(56.8)	4,158(58.0)	5,758(59.5)	6,940(60.0)	3.96	< 0.001
	no	14,455(41.1)	2,905(43.2)	3,005(42.0)	3919(40.5)	4,626(40.0)		
Missing	Yes	1,005(2.8)	304(4.3)	323(4.3)	206(2.1)	172(1.5)	−12.15	< 0.001
	no	35,131(97.2)	6,725(95.7)	7,163(95.7)	9,677(97.9)	11,566(98.5)		
**Masking**								
None	Yes	20,426(56.9)	3,923(56.6)	4,116(55.4)	5,765(58.6)	6,622(56.5)	0.84	0.404
	no	15,490(43.1)	3,003(43.4)	3,318(44.6)	4,071(41.4)	5,098(43.5)		
Missing	Yes	220(0.6)	103(1.5)	52(0.7)	47(0.5)	18(0.2)	−9.52	< 0.001
	no	35,916(99.4)	6,926(98.5)	7,434(99.3)	9,836(99.5)	11,720(99.8)		
**Allocation**								
Randomized	Yes	23,643(66.0)	4,618(66.8)	4,976(66.9)	6,387(64.9)	7,662(65.7)	−1.93	0.054
	no	12,196	2,292(33.2)	2,458(33.1)	3,447(35.1)	3,999(34.3)		
Missing	Yes	297(0.8)	119(1.7)	52(0.7)	49(0.5)	77(0.7)	−6.02	< 0.001
	no	35,839(99.2)	6910(98.3)	7434(99.3)	9,834(99.5)	11,661(99.3)		
**Lead sponsor**								
Industry	Yes	7,101(19.7)	1,909(27.2)	1,589(21.2)	1,779(18.0)	1,824(15.5)	−17.12	< 0.001
	no	29,035(80.3)	5,120(72.8)	5,897(78.8)	8,104(82.0)	9,914(84.5)		
NIH	Yes	469(1.3)	166(2.4)	102(1.4)	95(1.0)	106(0.9)	−7.29	< 0.001
	no	35,667(98.7)	6,863(97.6)	7,384(98.6)	9,788(99.0)	11,632(99.1)		
US federal	Yes	220(0.6)	66(0.9)	41(0.5)	74(0.7)	66(0.6)	−1.99	0.046
	no	35,916(99.4)	6,963(99.1)	7,445(99.5)	9,809(99.3)	11,672(99.4)		
Other	Yes	28,346(78.4)	4,888(69.5)	5,754(76.9)	7,962(80.6)	9,742(83.0)	19.02	< 0.001
	no	7,790(21.6)	2,141(30.5)	1,732(23.1)	1,921(19.4)	1,996(17.0)		

^a^ Cochran-Armitage test for temporal trend of four time subsets

As displayed in Table 1, the *Primary purpose* of trials oriented toward treatment comprised the largest proportion (60.0%) of interventional trials listed, while the proportion showed a decreasing trend over time (*Z* = − 16.12, *p* <  0.001). With the *Intervention* type, drug trials accounted for the largest proportion of trials (36.9% vs 17.1% for behavioral trials), showing a decreasing trend over time (from 48.8 to 28.9%) (*Z* = − 24.68, *p* <  0.001). Behavioral trials, conversely, increased from 12.6 to 20.4% (*Z* = 12.28, *p* <  0.001). The similar trend existed in observational studies.

For the scale of *Enrollment*, most interventional trials were small-scale (enrolling 1–100 participants) and showed an increasing trend over time (*Z* = 3.96, *p* <  0.001). As is presented in eTable 3–4, 93.1% of interventional trials, compared with 80.7% of observational studies, had 1000 or fewer participants. And 58.9% of interventional trials had 100 or fewer participants, while observational studies was 40.7%.

It could also be proved from Table 1 that most key variables were involved with *Missing* data. Whereas, the proportion of missing data has declined all the way through the study period. Specifically, from the first time subset (2008–2010) to the last time subset (2017–2019), the proportion of registered interventional trials that did not report a *Primary purpose* decreased from 261 (3.7%) to 0 (0.0%) (*Z* = − 15.90, *p* <  0.001); those that did not report *Enrollment* dropped from 304 (4.3%) to 172 (1.5%) (*Z* = − 12.15, *p* <  0.001); those that did not report *Masking* reduced from 103 (1.5%) to 18 (0.2%) (*Z* = − 9.25, *p* <  0.001); and those that did not report *Allocation* fell from 119 (1.7%) to 77 (0.7%) (*Z* = − 6.02, *p* <  0.001). The same downward trend of missing data were existed in observational studies about *Time perspective* (declined from 6.1 to 0.1%) (*Z* = − 14.97, *p* <  0.001) and *Observational model* (declined from 12.8 to 0.1%) (*Z* = − 23.36, *p* <  0.001, eTable 5).

Overall, interventional trials registered in ClinicalTrials.gov were dominated by small-scale trials and reported use of randomization, non-masking. And observational studies were dominated by prospective cohort studies.

### Lead sponsor and funding sources

For *Lead sponsor* of interventional trials, the proportion of trials reporting industry as lead sponsor decreased from 1,909 (27.2%) to 1,824 (15.5%) (*Z* = − 17.12, *p* <  0.001) during the four time subsets (Table 1). Meanwhile, the proportion of trials reporting NIH as lead sponsor descended from 166(2.4%) to 106 (0.9%) (*Z* = − 7.29, *p* <  0.001) and the proportion of trials reporting other lead sponsors rose from 4,888 (69.5%) to 9,742 (83.0%) (*Z* = 19.02, *p* <  0.001).

About probable funding sources, data on funding sources and funding sites were available for 31,902 of 36,136 interventional trials registered during the 2008–2019 period (Table 2). The largest proportion (21,394, 67.1%) of these trials were funded neither by industry (7,867, 24.6%) nor by the NIH (2,641, 8.3%) (Table 3). 69.5% (22,176) of trials were single-site and 30.5% (31,902) were multisite trials (Table 4). The largest proportion of trials (17,734, 39%) comprised single-site trials that funded neither by the NIH nor by industry and were typically small (62.8% had enrolled 1–100 participants) (Table 2).

**Table 2:** Characteristics of interventional trials by funding sources ^a^ and funding sites, 2008–2019

	Trials, No.(%)					
	Industry-funded		NIH-funded		Other	
	Single-site (***n*** = 2,786)	Multisite (***n*** = 5,081)	Single-site (***n*** = 1,656)	Multisite (***n*** = 985)	Single-site (***n*** = 17,734)	Multisite (***n*** = 3,660)
**Status**						
Not yet recruiting	51(1.8)	27(0.5)	30(1.8)	12(1.2)	369(2.1)	85(2.3)
Recruiting	316(11.3)	718(14.1)	377(22.8)	204(20.7)	3,040(17.1)	902(24.6)
Enrolling by invitation	33(1.2)	57(1.1)	30(1.8)	12(1.2)	239(1.3)	43(1.2)
Active, not Recruiting	148(5.3)	502(9.9)	190(11.5)	169(17.2)	894(5.0)	316(8.6)
Completed	1,692(60.7)	3,125(61.5)	853(51.5)	499(50.7)	9,053(51.0)	1,694(46.3)
Suspended	12(0.4)	29(0.6)	19(1.1)	14(1.4)	108(0.6)	23(0.6)
Terminated	191(6.9)	427(8.4)	85(5.1)	43(4.4)	740(4.2)	158(4.3)
Withdrawn	76(2.7)	51(1.0)	30(1.8)	9(0.9)	435(2.5)	43(1.2)
Unknown status	267(9.6)	145(2.9)	42(2.5)	23(2.3)	2,856(16.1)	396(10.8)
**Primary purpose**						
Treatment	1,718(61.7)	3,867(76.1)	832(50.2)	646(65.6)	9,602(55.6)	2,096(58.4)
Prevention	458(16.4)	689(13.6)	363(21.9)	191(19.4)	3,008(17.4)	617(17.2)
Diagnostic	113(4.1)	80(1.6)	75(4.5)	19(1.9)	996(5.8)	181(5.0)
Other ^b^	393(14.1)	325(6.4)	364(22.0)	120(12.5)	3,656(21.2)	693(19.3)
Missing	104(3.7)	118(2.3)	23(1.4)	6(0.6)	472(2.7)	73(2.0)
**Intervention** ^**c**^						
Drug	1,281(42.1)	3,356(66.0)	463(27.8)	490(49.4)	5,189(29.3)	1,243(34.0)
Behavioral	132(4.5)	38(0.8)	735(44.5)	229(23.6)	3,583(20.2)	758(20.7)
Device	468(15.3)	455(8.9)	86(5.2)	56(5.8)	2,342(13.2)	386(10.5)
Biological	450(14.7)	1,020(20.0)	143(8.7)	130(13.5)	784(4.4)	236(6.4)
Other ^d^	692(23.2)	646(13.2)	587(36.4)	384(40.8)	7,643(44.2)	1,435(40.3)
**Enrollment**						
1–100	1,691(60.9)	2,487(49.0)	930(56.2)	427(43.4)	11,088(62.8)	1,692(47.0)
101–1,000	881(31.7)	2,208(43.5)	568(34.3)	454(46.1)	5,105(28.9)	1,491(41.4)
>1,000	134(4.8)	330(6.6)	126(7.6)	95(9.6)	1,022(5.8)	417(11.6)
Missing	70(2.5)	50(1.0)	30(1.8)	9(0.9)	440(2.5)	42(1.2)
**Interventional model**						
Single-group	1,077(38.7)	1,892(37.2)	467(28.2)	275(27.9)	4,695(26.5)	888(24.3)
Parallel	1,455(52.2)	2,820(55.5)	1,000(60.4)	630(64.0)	11,289(63.7)	2,387(65.2)
Other ^e^	242(8,7)	325(6.4)	181(11.0)	79(8.0)	1,660(9.4)	354(9.7)
Missing	12(0.4)	44(0.9)	8(0.5)	1(0.1)	90(0.5)	31(0.8)
**Masking**						
Non-masking	1,596(57.6)	2,939(58.0)	988(59.7)	606(61.5)	9,859(55.9)	2,213(60.9)
Masking	1,177(42.4)	2,126(42.0)	657(40.3)	375(38.5)	7,763(44.1)	1,418(39.1)
Missing	13(0.5)	16(0.3)	11(0.7)	4(0.4)	112(0.6)	29(0.8)
**Allocation**						
Randomized	1,596(57.6)	2,758(54.9)	1,094(66.1)	647(65.7)	12,085(68.6)	2,517(69.4)
Non-randomized	283(10.2)	713(14.2)	164(9.9)	104(10.6)	1,766(10.0)	381(10.5)
N/A	889(32.2)	1,550(30.8)	385(23.2)	229(23.2)	3,756(21.3)	728(20.1)
Missing	18(0.6)	60(1.2)	13(0.8)	5(0.5)	127(0.7)	34(0.9)
**Phase**						
0–2	1,004(36.1)	1,941(38.3)	554(33.5)	455(46.2)	3,467(19.6)	893(24.4)
3–4	820(29.3)	2,482(48.7)	127(7.6)	193(19.6)	2,697(15.2)	702(19.2)
N/A	962(34.6)	668(13.0)	975(58.9)	337(34.2)	11,570(65.2)	2,065(56.4)

^a^The trial funding sources was determined using the algorithm

^b^Includes supportive care, screening, health services research, basic science, and other

^c^Percentages may not sum to 100% as categories are not mutually exclusive

^d^Includes procedure, dietary supplement, radiation, Genetic, and other

^e^Includes crossover, factorial and sequential

Note: excludes 4,234 trials (11.7%) with missing data on facility location

**Table 3:** Characteristics of interventional trials by funding sources, 2008–2019

	Trials, No.(%)			*χ*^2^	***P*** value
	Industry-funded (***n*** = 7,867)	NIH-funded (***n*** = 2,641)	Other (***n*** = 21,394)		
**Founding sites**				6,181.2	< 0.001
Single-site	2,786(35.4)	1,656(62.7)	17,734(82.9)		
Multisite	5,081(64.6)	985(37.3)	3,660(17.1)		
**Status_Completed**				283.1	< 0.001^a^
Yes	4,817(61.2)	1,352(51.2)	10,747(50.2)		
No	3,050(38.8)	1,289(48.8)	10,647(49.8)		
**Primary purpose_treatment**				641.6	< 0.001^b^
Yes	5,585(71.0)	1,478(56.0)	11,698(54.7)		
No	2,282(29.0)	1,163(44.0)	9,696(45.3)		
**Intervention_Drug**				4,043.9	< 0.001
Yes	4,637(58.9)	964(36.5)	4,341(20.3)		
No	3,230(41.1)	1,677(63.5)	17,053(79.7)		
**Phase_phase 3–4**				2,296.7	< 0.001
Yes	3,230(41.1)	320(12.1)	3,399(15.9)		
No	4,637(58.9)	2,321(87.9)	17,995(84.1)		

*χ*^2^ test for effect of funding sources (industry, NIH, or other) influencing proportion of trials

^a^Multiple comparisons: NIH-funded vs Other adjusted *p* value = 0.360, *p* <  0.001 for those not specifically noted (adjustment method: fdr)

^b^Multiple comparisons: NIH-funded vs Other adjusted *p* value = 0.220, *p* <  0.001 for those not specifically noted (adjustment method: fdr)

**Table 4:** Characteristics of interventional trials by funding sites, 2008–2019

	Trials, No.(%)		*χ*^2^	***P*** value
	Single-site (***n*** = 22,176)	Multisite (***n*** = 9,726)		
**Enrollment**			672.5	< 0.001^a^
1–100	13,709(63.6)	4,606(48.0)		
101–1,000	6,554(30.4)	4,153(43.2)		
>1,000	1,282(6.0)	842(8.8)		
**Primary purpose_Treatment**			482.9	< 0.001
yes	12,152(54.8)	6,609(68.0)		
no	10,024(45.2)	3,117(32.0)		
**Intervention_Drug**			1,276.9	< 0.001
yes	6,933(31.3)	5,089(52.3)		
no	15,243(68.7)	4,637(47.7)		

*χ*^2^ test for effect of funding sites (Single-site, Multisite) influencing proportion of trials

^a^Multiple comparisons: 101–1,000 vs > 1,000 adjusted *p* value = 0.480, *p* <  0.001 for those not specifically noted (adjustment method: fdr)

Table 4 showed that, for *Status* of funding sources, trials funded by industry comprised the largest proportion of trials listed as currently completed: 61.2% vs 51.2% for trials funded by NIH and 50.2% for trials funded by other (*χ*^2^ = 283.1, *p* <  0.001). Likewise, about *Primary purpose* and *Phase*, trials funded by industry possessed the largest proportion toward treatment (71.0% vs 56.0% for NIH and 54.7% for other) (*χ*^2^ = 641.6, *p* <  0.001), being more oriented toward later-phase research (i.e, phase 3 and 4) (*χ*^2^ = 2,296.7, *p* <  0.001). For *Intervention* type, 58.9% of trials funded by industry evaluated drugs, followed by 36.5% of trials funded by NIH and 20.3% of trials funded by other (*χ*^2^ = 4,043.9, *p* <  0.001).

### Pediatric clinical conditions

With temporal trend, the proportion of five conditions (infectious, cancer, immune, respiratory and digestive) trials showed a decreasing trend from the first time-subset (2008–2010) to the last time subset (2017–2019) (eTable 6). In contrast, the proportion of mental trials presented an increasing trend over time, from 605 (8.6%) to 1,292 (11.0%) (*Z* = 5.08, *p* <  0.001). In addition, for observational studies, the proportion of mental studies as well as digestive studies had no significant temporal trend (eTable 7).

According to Table 5, the critical trial characteristics (*Status*, *Study design*, *Enrollment* and *Phase*) of interventional trials were selected to examine heterogeneity among top four pediatric clinical conditions (infectious, cancer, immune, and respiratory).

**Table 5:** Characteristics and study designs of interventional trials by conditions, 2008–2019

		Trials, No.(%)				*χ*^2^	***P*** value
		Infectious (***n =*** 4,863)	Cancer (***n*** = 4,389)	Immune (***n*** = 4,288)	Respiratory (***n*** = 3,855)		
**Status**							
Recruiting	Yes	**537(18.3)**	**1,183(40.4)**	**751(25.6)**	**459(15.7)**	505.6	< 0.001^a^
	No	4,326(30.0)	3,206(22.2)	3,537(24.5)	3,396(23.5)		
Completed	Yes	**3,064(33.6)**	**1,480(16.2)**	**2,274(24.9)**	**2,313(25.3)**	923.4	< 0.001^b^
	No	1,799(21.8)	2,909(35.2)	2,014(24.4)	1,542(18.7)		
**Primary purpose**							
Treatment	Yes	**2,179(20.5)**	**3,167(29.8)**	**3,009(28.3)**	**2,272(21.4)**	1,355.7	< 0.001^c^
	No	2,612(47.6)	1,171(21.3)	1,194(21.8)	510(9.3)		
Prevention	Yes	**1,822(55.1)**	**295(8.9)**	**472(14.3)**	**716(21.7)**	1,689.5	< 0.001
	No	2,969(21.5)	4,043(29.3)	3,731(27.0)	3,066(22.2)		
Missing		72	51	85	73		
**Enrollment**							
1–100	Yes	1,590(17.5)	3,037(33.4)	2,576(28.3)	1,902(20.9)	1,439.5	< 0.001
	No	3,114(40.5)	1,189(15.5)	1,569(20.4)	1,819(23.7)		
101–1000	Yes	2,267(36.4)	1,063(17.1)	1,386(22.2)	1,515(24.3)	551.2	< 0.001
	No	2,437(23.1)	3,163(29.9)	2,759(26.1)	2,206(20.9)		
>1000	Yes	847(58.0)	126(8.6)	183(12.5)	304(20.8)	784.6	< 0.001
	No	3,857(25.1)	4,100(26.7)	3,962(25.8)	3,417(22.2)		
Missing		159	126	183	304		
**Gender**							
Female only	Yes	283(34.6)	405(49.5)	84(10.3)	46(5.6)	392.3	< 0.001^d^
	No	4,572(27.6)	3,973(24.0)	4,195(25.4)	3,801(23.0)		
Male only	Yes	71(20.1)	206(58.2)	63(17.8)	14(3.9)	224.8	< 0.001
	No	4,784(28.1)	4,172(24.5)	4,216(24.8)	3,833(22.5)		
Both		4,501	3,767	4,132	3,787		
Missing		8	11	9	8		
**Interventional model**							
Single-group	Yes	**1,081(18.4)**	**2,421(41.2)**	**1,517(25.8)**	**8,56(14.6)**	1,472.0	< 0.001^e^
	No	3,761(33.0)	1,898(16.7)	2,746(24.1)	2,993(26.3)		
Parallel	Yes	**3,506(34.8)**	**1,689(16.8)**	**2,302(22.8)**	**2,586(25.6)**	1,208.9	< 0.001
	No	1,336(18.6)	2,630(36.6)	1,961(27.3)	1,263(17.6)		
Missing		21	70	25	6		
**Masking**							
Masking	Yes	2,166(34.6)	668(10.7)	1,511(24.2)	1,931(30.5)	1,275.6	< 0.001
	No	2,676(24.2)	3,678(33.3)	2,755(25.0)	1,911(17.5)		
Missing		21	43	22	13		
**Allocation**							
Randomized	Yes	3,511(34.4)	1,481(14.5)	2,417(23.6)	2,810(27.5)	532.33	< 0.001
	No	516(23.6)	742(33.9)	570(26.0)	362(16.5)		
N/A		816	2,095	1,258	669		
Missing		20	71	43	14		
**Phase**							
3–4	Yes	2,017(39.0)	575(11.2)	1,229(23.8)	1,345(26.0)	1427.8	< 0.001^f^
	No	1,277(20.3)	2,471(39.3)	1,683(26.8)	859(13.7)		
NA		1,569	1,343	1,376	1,651		

*χ*^2^ test for effect of conditions (infectious, caner, immune, respiratory) influencing proportion of trials.

^a^Multiple comparisons: Infectious vs Respiratory adjusted *p* value = 0.220, *p* < 0.001 for those not specifically noted (adjustment method: fdr)

^b^Multiple comparisons: Infectious vs Respiratory adjusted *p* value = 0.004

^c^Multiple comparisons: Cancer vs Immune adjusted *p* value = 0.150

^d^Multiple comparisons: Immune vs Respiratory adjusted *p* value = 0.007

^e^Multiple comparisons: Infectious vs Respiratory adjusted *p* value = 0.940

^f^Multiple comparisons: Infectious vs Respiratory adjusted *p* value = 0.900

On *Status* and *Study design*, of these four categories, infectious trials were most numerous (*n* = 4,863), accounting for the largest proportion of trials listed as completed: 3,064 (33.6%) vs 1,480 (16.2%) for cancer, 2,274 (24.9%) for immune and 2,313 (25.3%) for respiratory trials (*χ*^2^ = 923.4, *p* <  0.001). Infectious trials also formed the largest proportion of trials oriented toward prevention: 1,822 (55.1%) vs 295 (8.9%) for cancer, 472 (14.3%) for immune and 716 (21.7%) for respiratory (*χ*^2^ = 1,689.5, *p* <  0.001). Among treatment-oriented trials, cancer trials comprised the largest group: 3,167 (29.8%) vs 2,179 (20.5%) for infectious, 3,009 (28.3%) for immune and 2,272 (21.4%) for respiratory (*χ*^2^ = 1,355.7, *p* <  0.001). Moreover, cancer trials accounted for the largest proportion of trials listed as recruiting: 2,272 (40.4%) vs 537 (18.3%) for infectious, 751 (25.6%) for immune and 459 (15.7%) for respiratory (*χ*^2^ = 505.6, *p* <  0.001). Also, cancer trials were more likely to involve a single-group of participants with randomization of treatment assignment: 2,421 (41.2%) vs 1,517 (25.8%) for immune, 1,081 (18.4%) for infectious and 856 (14.6%) for respiratory (*χ*^2^ = 1,472.0, *p* <  0.001). And most of cancer trials were small-scale and non-masking. Infectious trials, on the other side, were more likely to utilize parallel-group design with randomization of prevention assignment: 3,506 (34.8%) vs 2,586 (25.6%) for respiratory, 2,302 (22.8%) for immune and 1,689 (16.8%) for cancer (*χ*^2^ = 1,208.9, *p* <  0.001).

For *Enrollment* and *Phase*, infectious trials made up the largest proportion of trials that were medium-scale (enrollment between 101 and 1,000, 36.4%) and large-scale (enrollment greater than 1,000, 58.0%). Infectious and respiratory trials were usually oriented toward later-phase research (i.e, phase 3 and 4), while cancer and immune trials tended toward earlier-phase trials (i.e, early phase 1 through phase 2).

### Proportion of reporting study results

About overall situation of reporting study results, 88.5% (46,935/53,060) of registered studies (from January 2008 to December 2019) did not report results, presenting 95.7% (15,970/16,692) of the observational studies and 85.0% (30,733/36,136) of the interventional trials (eTable 8). Based on Table 6, interventional trials, compared with observational studies, had the largest proportion of reporting results: 5,403 (15.0%) vs 722 (4.3%) (*χ*^2^ = 1,256.8, *p* <  0.001). For lead sponsor, studies sponsored by industry had higher proportion of reporting results: 132 (15.8%) vs 3,080 (30.8%) for NIH (*χ*^2^ = 82.9, *p* <  0.001), 52 (20.2%) vs 3,080 (30.8%) for US federal (*χ*^2^ = 12.9, *p* <  0.001) and 2,861 (6.9%) vs 3,080 (30.8%) for other (*χ*^2^ = 4,547.6, *p* <  0.001). About *Enrollment*, studies with 1,000 or more participants had lower proportion of reporting results: 528 (9.5%) vs 3,349 (11.7%) for enrollment of 1–100 (*χ*^2^ = 22.8, *p* <  0.001) and 528 (9.5%) vs 2,248 (12.1%) for enrollment of 101–1,000 (*χ*^2^ = 28.9, *p* <  0.001). With *Phase*, the studies oriented toward later-phase research(i.e, phase 3 and 4) shared higher proportion of reporting results: 2,204 (56.3%) vs 1,645 (42.7%) for early phase 1- phase 2 (*χ*^2^ = 218.8, *p* <  0.001).

**Table 6:** Proportion of reporting results within all studies by characteristics, 2008–2019

	Trials, No.(%)		*χ*^2^	***P*** value
	Has Results (***n*** = 6,125)	No Results Available (***n*** = 46,935)		
**Study type**			1,256.8	< 0.001
Interventional	**5,403(15.0)**	30,733(85.0)		
observational	**722(4.3)**	15,970(95.7)		
**Lead sponsor**			4,545.6	< 0.001^a^
Industry	**3,080(30.8)**	6,920(69.2)		
NIH	**132(15.8)**	705(84.2)		
US federal	**52(20.2)**	206(79.8)		
Other	**2,861(6.9)**	38,872(93.1)		
**Enrollment**			29.4	< 0.001^b^
1–100	**3,349(11.7)**	25,170(88.3)		
101–1,000	**2,248(12.1)**	16,259(87.9)		
> 1,000	**528(9.5)**	5,025(90.5)		
**Phase**			218.8	< 0.001
Early Phase 1-Phase 2	**1,645(42.7)**	7,413(57.3)		
Phase 3-Phase 4	**2,204(56.3)**	5,759(43.7)		

*χ*^2^ test for effect of characteristics influencing proportion of reporting study results

^a^Multiple comparisons: NIH vs US adjusted *p* value = 0.120, *p* < 0.001 for those not specifically noted (adjustment method: fdr)

^b^Multiple comparisons: 1–100 vs 101–1,000 adjusted *p* value = 0.190

**Fig. 1:**
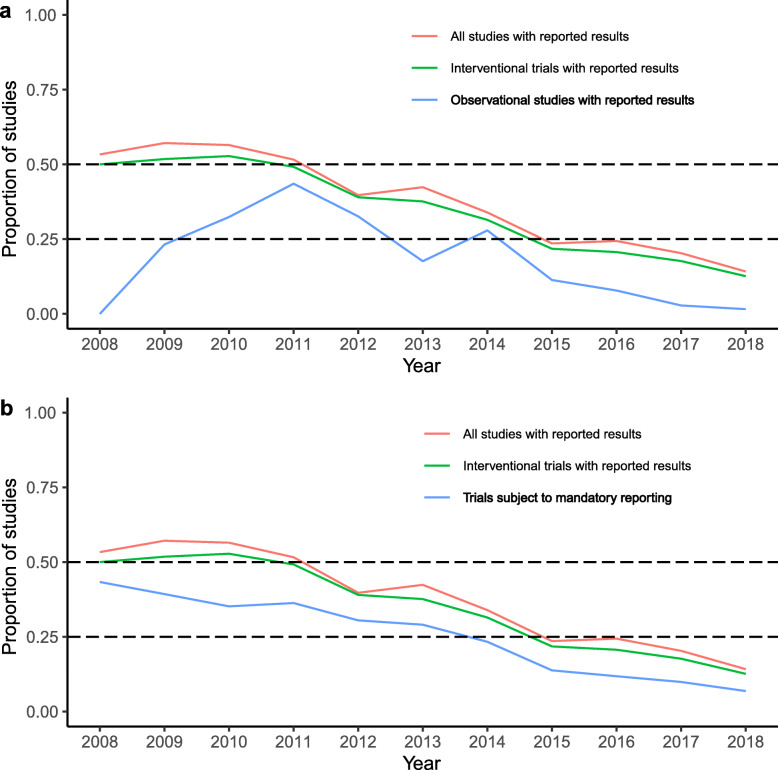
Proportion of three type studies with reported results within all completed studies. **a** Includes all studies, interventional trials and observational studies. **b** Includes all studies, interventional trials and trials subject to mandatory reporting

Fig. 1 displayed the proportion of studies including different study types (all studies, interventional trials, observational studies) with reported results within all completed studies per year from 2008 through 2018. From 2008 to 2018, 21.4% (1,679/7,838) of all completed studies have reported results: from 53.3% (60/30) to 14.2% (441/3,111), with the highest proportion (57.1%, 32/56) in 2009 (Fig. 1a). Meanwhile, for interventional trials, 19.1% (1,494/7,838) have reported results: from 50.0% (15/30) to 12.6% (392/3,111), with the highest proportion (52.8%, 57/108) in 2010. As to observational studies, 6.5% (510/7,838) have reported results: the highest proportion was 43.5% (54/124) in 2011. On the whole, the proportion distribution of observational studies with results reported was lower than those of interventional trials. And the proportion distribution of interventional trials with results reported existed similar trend with all studies.

**Fig. 2:**
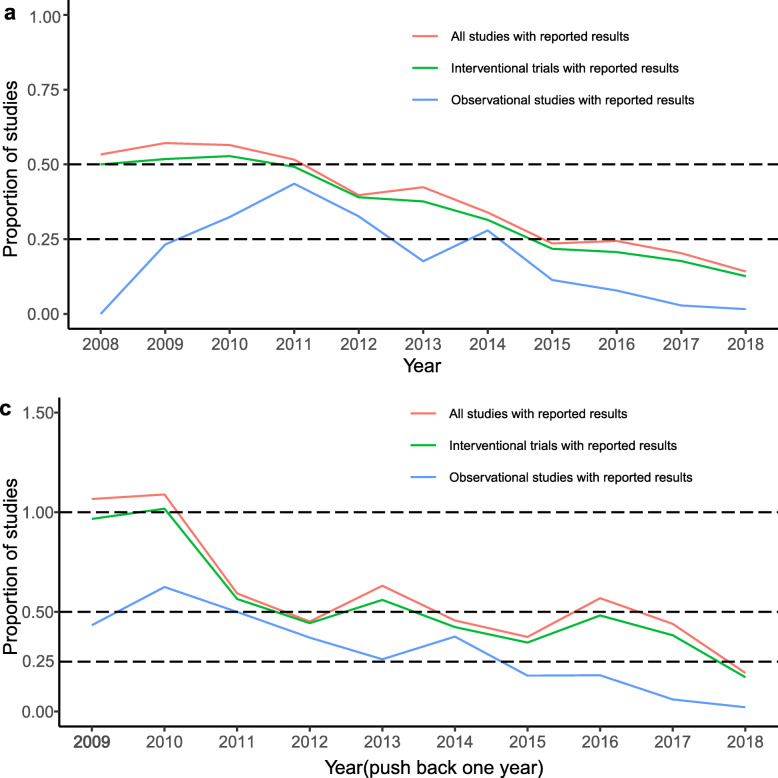
Proportion of three type studies with reported results within all completed studies (push back one year). **a** Same as Fig. 1**a**. **c** The completion date of studies had been pushed back one year

For ‘trials subject to mandatory reporting’, 11.6% (912/7,838) have reported results: from 43.3% (13/30) to 6.8% (213/3,111), having the highest proportion (39.3%, 22/56) in 2009 and presenting the similar trend with interventional trials and all studies. This may suggest that before policies requiring completed trials to report results took effect on a large scale, ‘trials subject to mandatory reporting’ may formed a major part of trials with results reported (Fig. 1b). Further, the proportion distribution of observational studies was in a low position and presented irregular changes, might attributing to observational studies beyond general scope of policy requirements (Fig. 1a). As in Fig. 2, after pushing back the completion date of the studies by 1 year, the proportion distribution of three type studies had generally been improved (Fig. 2a vs c). However, it was worth noting that the overall proportion remained below 50%.

## Discussion

This article provided a preliminary outline of clinical registration studies in the pediatric field over the past 12 years, and there were a few results that might deserve further attentions.

First, the pediatric clinical registration studies in this article were studies related to children, without strictly excluding adults. Those studies were dominated by small-scale, single-site trials and were funded neither by the NIH nor by industry. Due to the different age criteria for selecting research studies, our results may differ from studies containing only children. Previous findings embodied that RCTs (Randomized Controlled Trials), recruiting children and adults simultaneously, were more likely to complete trials than those recruiting only adults [18]. According to the Supplementary analysis of this study (eTable 9–11), consistent with previous findings, trials including only children had the higher proportion of completed status as well as behavioral interventions, comparing to trials containing both children and adults. The data suggested that behavioral interventions were playing an increasingly important role in pediatric clinical trials. Meanwhile, mental trials had higher proportion of behavioral interventions than other four common pediatric conditions (infectious, cancer, immune, respiratory) (*χ*^2^ = 3,696.1, *p* <  0.001), with no significant difference after classifying the children-only subgroup (eTable 11, *χ*^2^ = 0.8, *p* = 0.378). This may indicated that behavioral interventions were gradually adopted in the field of child mental health. As of 2001, the World Health Organization reported that one in four people worldwide suffered from a mental health disorder during their lifetime, and 46.6 million people in the United States suffered from a mental health disorder [19]. The field of mental health has begun to utilize ClinicalTrials.gov to help answer some questions regarding its clinical trials [10, 20].

Second, this study not only concerned about the interventional trials, but also analyzed observational studies. An increasing temporal trend in registered observational studies could be observed: from 3,116 (30.7%) to 6,156 (34.4%) (*Z* = 5.75, *p* <  0.001) (eTable 5). The registration of observational studies remained controversial, although the registration of randomized trials had been widely accepted. For example, previous studies have shown that registration of observational studies cannot effectively prevent false positive results in observational studies [21]. Some of these studies were registered after their results were published, but post-publication registration was more liable to publication or reporting bias, since researchers could selectively register research ideas after the data has been explored [22]. The fully reporting of research methods and results in a timely and impartial manner was essential for realizing research benefits [23]. Although the registration of observational studies was still controversial, the observational studies, especially small-scale and medium-scale prospective cohort studies, still presented an increasing trend.

Last but not least, we also focused on the proportion of reporting study results. Given that policy required trials to report results, the quality of results reported of existing pediatric registered trials was still poor and had a room for improvement. Previous studies had found that pediatric clinical trials were frequently discontinued or the results were not published. Thousands of children had participated in these trials, representing considerable inefficiencies and waste of financial and human resources [24]. This should arouse our attention regarding the overall standards of pediatric clinical trials reporting. This article found that compared with interventional trials, observational studies had lower proportion of reporting results. The FDA and the final rule did not mandate that all studies report their results to the registry, which may be the reason that few studies had results reported [25] (eTable 1). Besides, the final rule, effective on January 18, 2017, expanded the scope of trials that must report results. Clinical trials registered before policies implementation were less likely to report results, which could be observed in our analysis and previous study [26]. In addition to completion date, lead sponosr and intervention types were also associated with results reporting. It is known that non-selective dissemination of research results was critical to clinical practice. As mentioned in the results, trials sponsored by industry had higher proportion of reporting results. A previous study had explained that industry sponsors tended to be well-staffed and had a centralized process to support the submission of results, while non-industry sponsors tended to rely on a single investigator with very little centralized support [27]. Therefore, how to improve the quality of results reporting while giving consideration to the entire life cycle of clinical trials may be a critical issue to be considered in the future.

Several limitations of our study should be noted. First, our study focused on clinical registration studies related to children, and rigorous analysis of pediatric clinical registration trials may divide child-only registration trials into a separate analysis for comparison. Second, not all trials, for example phase 1 trials or trials looking at non-drug interventions, met FDA or final rule requirements [28]. There may be other incentives and norms that bias the registration of trials with certain characteristics. In this case, trends identified by registries reflect at least partial changes in trial reporting rather than changes in how trials are conducted or designed. Finally, the studies which did not register on ClinicalTrials.gov were not included in our evaluation.

## Conclusions

Through examining temporal trend and heterogeneity among subgroups, the study updated and summarized the changes of pediatric clinical studies registered in ClinicalTrials.gov, 2008–2019. There was significant heterogeneity in trial characteristics among subgroups including funding sources, pediatric clinical conditions and proportion of reporting results. To provide the public with more transparent and high-quality pediatric clinical study information, future researches need to improve the recommendations for registration information as well as reporting results.

## Supplementary Information

**Additional file 1.**

## Data Availability

The data sets generated and analyzed during the current study are available in the Clinical Trials.gov website repository, http://clinicaltrials.gov/

## Abbreviations

AACT: Aggregate Analysis of ClinicalTrials.gov
NA: Not Applicable
NLM: National Library of Medicine
NIH: National Institutes of Health
FDAAA: Food and Drug Administration Amendments Act
RCTs: Randomized Controlled Trials
