# Supplementary material for: Updated analysis of pediatric clinical studies registered in ClinicalTrials.gov, 2008–2019
Source: BMC Pediatr. 2021 Apr 30;21:212. doi: 10.1186/s12887-021-02658-4 (PMC8086350; doi:10.1186/s12887-021-02658-4)
Supplement: Supplementary file 1 — Additional file 1. [file 12887_2021_2658_MOESM1_ESM.docx]

**Supplementary content**

eTable 1 Policies regarding reporting of clinical study results in the history of ClinicalTrials.gov.

eTable 2 Data elements for studies registered at ClinicalTrials.gov.

eTable 3 Characteristics for interventional trials registered in ClinicalTrials.gov, 2008-2019

eTable 4 Characteristics for observational studies registered in ClinicalTrials.gov, 2008-2019

eTable 5 Temporal trend for characteristics of observational studies registered in ClinicalTrials.gov, 2008-2019

eTable 6 Temporal trend for condition categories of interventional trials registered in ClinicalTrials.gov, 2008-2019

eTable 7 Temporal trend for condition categories of observational studies registered in ClinicalTrials.gov, 2008-2019

eTable 8 Characteristics of reporting results within different study types, 2008-2019

eTable 9-11 Supplementary analysis

eTable 1 Policies regarding reporting of clinical study results in the history of ClinicalTrials.gov [27].

| **Name** | **General Scope** | **Submission Type and Timeline** | **Relevant Dates** |
| --- | --- | --- | --- |
| International Committee of Medical  Journal Editors Clinical Trial  Registration Policy | Clinical trials of any type  of intervention  Includes phase 1 trials and  small feasibility studies | **Registration:** On or before enrollment of first trial participant  **Summary results:** If required, at time specified by funding and regulatory agencies; otherwise encouraged | **Effective dates:**  Clinically directive clinical trials initiated on or after July 1, 2005  Ongoing clinically directive clinical trials initiated before September 13, 2005  All clinical trials (expanded to include phase 1) initiated on or after July 1, 2008 |
| FDA Amendments Act (FDAAA)  (U.S. federal law) and associated  regulations, 42 CFR Part 11 (U.S.  federal regulations) | Clinical trials of FDA regulated drug, biologic, or device products (“applicable clinical trials”)  Excludes phase 1 trials and small feasibility studies | **Registration:** Within 21 days after enrollment of the first trial participant  **Summary results:** Within 12 months after the primary completion date; submission may be delayed with certification, if specific conditions are met, for up to 2 additional years or delayed for an approved period with extension request for “good cause” | **FDAAA enacted on September 27, 2007**  **Registration:** Applicable clinical trials initiated after September 27, 2007, or initiated on or before that date but still ongoing as of December 26, 2007  **Summary results:** Applicable clinical trials of FDA-approved, licensed, or cleared products that reached a “primary completion date” (i.e., data collection complete for the primary outcome) after December 26, 2007 |
|  |  |  | **Regulatory** **effective date: January 18, 2017**  **Registration:** Applicable clinical trials initiated on or after January 18, 2017  **Summary results:** Applicable clinical trials that reached their primary completion date on or after January 18, 2017, regardless of product approval, licensing, or clearance by FDA |
| Dissemination of NIH-funded clinical trial information6 (NIH policy) | NIH-funded clinical trials of  any type of intervention Includes phase 1 trials and  small feasibility studies | **Registration:** Same as for FDAAA  and 42 CFR 11  **Summary results:** Same as for FDAAA  and 42 CFR 11 | **Effective date:** January 18, 2017  Clinical trials supported by grants, other transactions, and contracts that submitted applications on or after January 18, 2017, for trials initiated on or after January 18, 2017  Clinical trials supported by NIH intramural program and initiated on or after January 18, 2017 |
| Submission of results of valid analyses by sex and race  (U.S. federal law and NIH policy) | NIH-funded, phase 3 clinical trials subject to FDAAA-associated regulations | **Registration:** Same as for FDAAA; outcomes specified by sex and race  **Summary results:** Same as for FDAAA; “valid analyses” specified by sex and race | Phase 3 applicable clinical trials (NIH-defined) under federal regulations supported by all new, competing grants and cooperative agreements awarded on or after December 13, 2017 |

eTable 2 Data elements for studies registered at ClinicalTrials.gov.

| **Variable Name** | **Value List** | **Definitions** |
| --- | --- | --- |
| Study Type | Interventional  Observational  Expanded access | **Interventional**: studies in human beings in which individuals are assigned by an investigator based on a protocol to receive specific interventions.  **Observationa**l: studies in human beings in which biomedical and/or health outcomes are assessed in pre-defined groups of individuals. Subjects in the study may receive diagnostic, therapeutic, or other interventions, but the investigator does not assign specific interventions to the subjects of the study.  **Expanded Access**: records describing the procedure for obtaining an experimental drug or device for patients who are not adequately treated by existing therapy, who do not meet the eligibility criteria for enrollment, or who are otherwise unable to participate in a controlled clinical study. |
| Status | Not yet recruiting  Active, not recruiting  Suspended  Withdrawn  Terminated  Completed  Enrolling by invitation  Recruiting  Unknown status | Overall accrual activity for the protocol. |
| Study Results | Has Results  No Results Available | A study record that includes the summary results posted in the ClinicalTrials.gov results database. |
| Condition |  | The disease, disorder, syndrome, illness, or injury that is being studied. On ClinicalTrials.gov, conditions may also include other health-related issues, such as lifespan, quality of life, and health risks. |
| Intervention | Drug  Procedure  Device  Biological  Radiation  Genetic  Other  Dietary Supplement  Behavioral | Type of intervention: Drug (including placebo), Device (including sham), Biological/Vaccine, Procedure/Surgery, Radiation, Behavioral (e.g., Psychotherapy, Lifestyle Counseling), Genetic (including gene transfer, stem cell and recombinant DNA), Dietary Supplement (e.g., vitamins, minerals), Other. |
| Lead Sponsor and Collaborators |  | Lead Sponsor: Name of primary organization that oversees implementation of study and is responsible for data analysis.  Examples: National Institute of Allergy and Infectious Diseases, Bristol-Myers Squibb.  Collaborator: Other organizations (if any) providing support, including funding, design, implementation, data analysis and reporting. The data provider is responsible for confirming all collaborators before listing them. |
| Gender | Both  Female  Male | Physical gender of individuals who may participate in the protocol. |
| Phase | N/A  Phase 4  Phase 3  Phase 2/Phase 3  Phase 2  Phase 1/Phase 2  Phase 1  Early Phase 1 | The stage of a clinical trial studying a drug or biological product, based on definitions developed by the U.S. Food and Drug Administration (FDA). |
| Enrollment |  | Target or Actual Number of Subjects. Number of subjects in the trial. |
| Study designs |  | The investigative methods and strategies used in the clinical study. It includes Allocation, Intervention/Observational model, Masking, Primary purpose, Time perspective. |
| Primary completion  date |  | The date on which the last participant in a clinical study was examined or received an intervention to collect final data for the primary outcome measure. Whether the clinical study ended according to the protocol or was terminated does not affect this date. For clinical studies with more than one primary outcome measure with different completion dates, this term refers to the date on which data collection is completed for all the primary outcome measures. The "estimated" primary completion date is the date that the researchers think will be the primary completion date for the study. |
| First posted |  | The date on which the study record was first available on ClinicalTrials.gov. There is typically a delay of a few days between the date the study sponsor or investigator submitted the study record and the first posted date. |
| Allocation | Randomized  Non-Randomized  N/A : single study | A method used to assign participants to an arm of a clinical study. The types of allocation are randomized allocation and non-randomized. |
| Intervention model | Single-group  Parallel  Crossover  Factorial  Sequential | The general design of the strategy for assigning interventions to participants in a clinical study. Types of intervention models include: single-group assignment, parallel assignment, cross-over assignment, and factorial assignment. |
| Masking | Open  Single  Double  Triple  Quadruple | A clinical trial design strategy in which one or more parties involved in the trial, such as the investigator or participants, do not know which participants have been assigned which interventions. Types of masking include: open label, single blind masking, and double-blind masking. |
| Primary purpose | Treatment  Prevention  Diagnostic  supportive care  Screening  health services research  basic science  other | The main reason for the clinical trial. The types of primary purpose are: treatment, prevention, diagnostic, supportive care, screening, health services research, basic science, and other. |
| Observational model |  | The general design of the strategy for identifying and following up with participants during an observational study. Types of observational study models include cohort, case-control, case-only, case-cross-over, ecologic or community studies, family-based, and other. |

eTable 3 Characteristics for interventional trials registered in ClinicalTrials.gov, 2008-2019

|  | **Trials, No.(%)** | | | | |
| --- | --- | --- | --- | --- | --- |
|  | **All,2008-2019**  (**n = 36,136)** | **2008-2010**  **(n = 7,029)** | **2011-2013**  **(n = 7,486)** | **2014-2016**  **(n = 9,883)** | **2017-2019**  **(n = 11,738)** |
| **Primary purpose** |  |  |  |  |  |
| Treatment | **21,126(60.0)** | **4,608(68.1)** | **4476(62.7)** | **5,614(58.7)** | **6,428(54.8)** |
| Prevention | **6,110(17.4)** | **1,227(18.1)** | **1376(19.3)** | **1,514(15.8)** | **1,962(16.7)** |
| Supportive Care | 1,938(5.5) | 264(3.9) | 360(5.0) | 583(6.1) | 731(6.2) |
| Other ^a^ | 6,026(17.1) | 669(9.9) | 922(12.9) | 1,854(19.4) | 2,612(22.3) |
| Missing | 936(2.6) | 261(3.7) | 352(4.7) | 318(3.2) | 5(0.0) |
| **Intervention ^b^** |  |  |  |  |  |
| Drug | **13,348(36.9)** | **3,427(48.8)** | **3,066(41.0)** | **3,457(35.0)** | **3,398(28.9)** |
| Behavioral | **6,194(17.1)** | **889(12.6)** | **1,171(15.6)** | **1,739(17.6)** | **2,395(20.4)** |
| Device | 4,325(12.0) | 570(8.1) | 709(9.5) | 1,457(14.7) | 1,589(13.5) |
| Procedure | 3,366(9.3) | 658(9.4) | 709(9.5) | 952(9.6) | 1,047(8.9) |
| Biological | 3,011(8.3) | 759(10.8) | 673(9.0) | 756(7.6) | 823(7.0) |
| Dietary supplement | 1,623(4.5) | 353(5.0) | 381(5.1) | 446(4.5) | 443(3.8) |
| Genetic | 191(0.5) | 33(0.5) | 37(0.5) | 48(0.5) | 73(0.6) |
| Radiation | 383(1.1) | 91(1.3) | 77(1.0) | 109(1.1) | 106(0.9) |
| Other ^c^ | 7,409(20.5) | 1,015(14.4) | 1412(18.9) | 1,015(14.4) | 2,757(23.5) |
| **Enrollment** |  |  |  |  |  |
| 1-100 | **20,614(58.9)** | **3,820(56.8)** | **4,158(58.1)** | **5,758(59.5)** | **6,940(60.0)** |
| 101-1,000 | 11,995(34.2) | 2,461(36.6) | 2,490(34.8) | 3,251(33.6) | 3,828(33.1) |
| ＞1,000 | 2,417(6.9) | 444(6.6) | 515(7.2) | 668(6.9) | 798(6.9) |
| Missing | 1,005(2.8) | 304(4.3) | 323(4.3) | 206(2.1) | 172(1.5) |
| **Masking** |  |  |  |  |  |
| None | **20,426(56.9)** | **3,923(56.6)** | **4,116(55.4)** | **5,765(58.6)** | **6,622(56.5)** |
| Single | 5,757(16.0) | 922(13.3) | 1,192(16.0) | 1,600(16.3) | 2,043(17.4) |
| Double | 3,523(9.8) | 681(9.8) | 655(8.8) | 881(9.0) | 1,306(11.1) |
| Triple | 2,427(6.8) | 486(7.0) | 567(7.6) | 599(6.1) | 775(6.6) |
| Quadruple | 3,783(10.5) | 914(13.2) | 904(12.2) | 991(10.1) | 974(8.3) |
| Missing | 220(0.6) | 103(1.5) | 52(0.7) | 47(0.5) | 18(0.2) |
| **Allocation** |  |  |  |  |  |
| Randomized | **23,643(66.0)** | **4,618(66.8)** | **4,976(66.9)** | **6,387(64.9)** | **7,662(65.7)** |
| Nonrandomized | 3,869(10.8) | 936(13.5) | 740(10.0) | 1,029(10.5) | 1,164(10.0) |
| N/A | 8,327(23.2) | 1,356(19.6) | 1,718(23.1) | 2,418(24.6) | 2,835(24.3) |
| Missing | 297(0.8) | 119(1.7) | 52(0.7) | 49(0.5) | 77(0.7) |
| **Lead sponsor** |  |  |  |  |  |
| Industry | **7,101(19.7)** | **1,909(27.2)** | **1,589(21.2)** | **1,779(18.0)** | **1,824(15.5)** |
| NIH | 469(1.3) | 166(2.4) | 102(1.4) | 95(1.0) | 106(0.9) |
| US federal | 220(0.6) | 66(0.9) | 41(0.5) | 74(0.5) | 66(0.6) |
| Other | 28,346(78.4) | 4,888(69.5) | 5,754(76.9) | 7,962(80.6) | 9,742(83.0) |

^a^ Includes diagnostic, screening, health services research, basic science, and other. ^b^ Percentages may not sum to 100% as categories are not mutually exclusive. ^c^ See eTable 2.

eTable 4 Characteristics for observational studies registered in ClinicalTrials.gov, 2008-2019

|  | **Trials, No.(%)** | | | | |
| --- | --- | --- | --- | --- | --- |
|  | **All**  **2008-2019**  **(n = 16,692)** | **2008-2010**  **(n = 3,116)** | **2011-2013**  **(n = 3,295)** | **2014-2016**  **(n = 4,125)** | **2017-2019**  **(n = 6,156)** |
| **Intervention ^a^** |  |  |  |  |  |
| Drug | **1,524(18.2)** | **346(24.9)** | **392(29.0)** | **377(16.8)** | **409(12.0)** |
| Behavioral | 377(4.5) | 60(4.3) | 55(4.1) | 81(3.6) | 181(5.3) |
| Device | **1,121(13.4)** | **136(9.8)** | **171(12.7)** | **305(13.6)** | **509(15.0)** |
| Procedure | 1,128(13.4) | 196(14.1) | 160(11.8) | 302(13.4) | 470(13.8) |
| Biological | 291(3.5) | 74(5.3) | 55(4.1) | 80(3.6) | 82(2.4) |
| Dietary supplement | 61(0.7) | 5(0.4) | 10(0.7) | 21(0.9) | 25(0.7) |
| Genetic | 312(3.7) | 121(8.7) | 71(5.3) | 50(2.2) | 70(2.1) |
| Radiation | 97(1.2) | 12(0.9) | 6(0.4) | 26(1.2) | 53(1.6) |
| Other ^b^ | 3,476(41.4) | 440(31.7) | 431(31.9) | 1,007(44.8) | 1,598(47.0) |
| **Enrollment** |  |  |  |  |  |
| 1-100 | **6,618(40.7)** | **1,185(40.5)** | **1,339(41.9)** | **1,487(36.8)** | **2,607(42.7)** |
| 101-1,000 | 6,512(40.0) | 1,175(40.1) | 1,252(39.1) | 1,712(42.4) | 2,373(38.9) |
| ＞1,000 | 3,136(19.3) | 569(19.4) | 608(19.0) | 840(20.8) | 1,119(18.3) |
| Missing | 426(2.6) | 187(6.0) | 96(2.9) | 86(2.1) | 57(0.9) |
| **Time perspective** |  |  |  |  |  |
| Prospective | **10,951(67.2)** | **2,055(70.2)** | **2,230(69.9)** | **2,824(69.9)** | **3,842(62.5)** |
| Retrospective | **2,768(17.0)** | **453(15.5)** | **554(17.4)** | **639(15.8)** | **1,122(18.2)** |
| Cross-Sectional | 1,985(12.2) | 349(11.9) | 327(10.3) | 461(11.4) | 848(13.8) |
| Other ^b^ | 602(3.7) | 69(2.4) | 78(2.4) | 117(2.9) | 338(5.5) |
| Missing | 386(2.3) | 190(6.1) | 106(3.2) | 84(2.0) | 6(0.1) |
| **Observational model** |  |  |  |  |  |
| Case-Control | 2,204(14.0) | 429(15.8) | 482(16.2) | 559(14.4) | 734(11.9) |
| Case-Crossover | 156(1.0) | 32(1.2) | 30(1.0) | 33(0.8) | 61(1.0) |
| Case-Only | **2,612(16.6)** | **604(22.2)** | **563(19.0)** | **547(14.1)** | **898(14.6)** |
| Cohort | 9,064(57.6) | 1,438(52.9) | 1,687(56.9) | 2,391(61.4) | 3,548(57.7) |
| Ecologic or Community | 300(1.9) | 58(2.1) | 52(1.8) | 67(1.7) | 123(2.0) |
| Family-Based | 187(1.2) | 59(2.2) | 26(0.9) | 46(1.2) | 56(0.9) |
| Other ^b^ | 1,202(7.6) | 98(3.6) | 127(4.3) | 248(6.4) | 729(11.9) |
| Missing | 967(5.8) | 398(12.8) | 328(10.0) | 234(5.7) | 7(0.1) |
| **Lead sponsor** |  |  |  |  |  |
| Industry | **2,899(17.4)** | **728(23.4)** | **785(23.8)** | **713(17.3)** | **673(10.9)** |
| NIH | 368(2.2) | 124(4.0) | 97(2.9) | 88(2.1) | 59(1.0) |
| US federal | 38(0.2) | 13(0.4) | 9(0.3) | 7(0.2) | 9(0.1) |
| Other | 13,387(80.2) | 2,251(72.2) | 2,404(73.0) | 3,317(80.4) | 5,415(88.0) |

^a^ Percentages may not sum to 100% as categories are not mutually exclusive. ^b^ See eTable 2.

eTable 5 Temporal trend for characteristics of observational studies registered in ClinicalTrials.gov, 2008-2019

|  |  | **Trials, No.(%)** | | | | | ***Z* value ^a^** | ***P* value** |
| --- | --- | --- | --- | --- | --- | --- | --- | --- |
|  |  | **All**  **2008-2019**  **(n = 1,6692)** | **2008-2010**  **(n = 3,116)** | **2011-2013**  **(n = 3,295)** | **2014-2016**  **(n = 4,125)** | **2017-2019**  **(n = 6,156)** |  |  |
| **Intervention** |  |  |  |  |  |  |  |  |
| Drug | Yes | 1,524(9.1) | 346(11.1) | 392(11.9) | 377(9.1) | 409(6.6) | -7.55 | <0.001 |
|  | no | 15,168(90.9) | 2,770(88.9) | 2,903(88.1) | 3,748(90.9) | 5,747(93.4) |  |  |
| Device | Yes | 1,121(6.7) | 136(4.4) | 171(5.2) | 305(7.4) | 509(8.3) | 6.92 | <0.001 |
|  | no | 15,571(93.3) | 2,980(95.6) | 3,124(94.8) | 3,820(92.6) | 5,647(91.7) |  |  |
| **Enrollment** |  |  |  |  |  |  |  |  |
| 1-100 | Yes | 6,618(40.7) | 1,185(40.5) | 1,339(41.9) | 1,487(36.8) | 2,607(42.7) | 1.24 | 0.2142 |
|  | no | 9,648(59.3) | 1,744(59.5) | 1,860(58.1) | 2,552(63.2) | 3,492(57.3) |  |  |
| Missing | Yes | 426(2.6) | 187(6.0) | 96(2.9) | 86(2.1) | 57(0.9) | -12.28 | <0.001 |
|  | no | 16,266(97.4) | 2,929(94.0) | 3,199(97.1) | 4,039(97.9) | 6,099(99.1) |  |  |
| **Time perspective** |  |  |  |  |  |  |  |  |
| Prospective | Yes | 10,951(67.3) | 2,055(70.2) | 2,230(69.9) | 2,824(70.5) | 3,842(62.5) | -7.07 | 0.404 |
|  | no | 5,322(32.7) | 871(29.8) | 959(30.1) | 1,184(29.5) | 2,308(37.5) |  |  |
| Retrospective | Yes | 2,768(17.0) | 453(15.5) | 554(17.4) | 639(15.9) | 1,122(18.2) | 2.46 | <0.001 |
|  | no | 13,505(83.0) | 2,473(84.5) | 2,635(82.6) | 3,369(84.1) | 5,028(81.8) |  |  |
| Mssing | Yes | 419(2.5) | 190(6.1) | 106(3.2) | 117(2.8) | 6(0.1) | -15.0 | <0.001 |
|  | no | 16,273(97.5) | 2,926(93.9) | 3,189(96.8) | 4,008(97.2) | 6,150(99.9) |  |  |
| **Observational model** |  |  |  |  |  |  |  |  |
| Cohort | Yes | 9,064(57.6) | 1,438(52.9) | 1,687(56.9) | 2,391(61.4) | 3,548(57.7) | 3.53 | <0.001 |
|  | no | 6,661(42.4) | 1,280(47.1) | 1,280(43.1) | 1,500(38.6) | 2,601(42.3) |  |  |
| Missing | Yes | 967(5.8) | 398(12.8) | 328(10.0) | 234(5.7) | 7(0.1) | -23.36 | <0.001 |
|  | no | 15,725(94.2) | 2,718(87.2) | 2,967(90.0) | 3,891(94.3) | 6,149(99.9) |  |  |
| **Lead sponsor** |  |  |  |  |  |  |  |  |
| Industry | Yes | 2,899(17.4) | 728(23.4) | 785(23.8) | 713(17.3) | 673(10.9) | -15.36 | <0.001 |
|  | no | 13,793(82.6) | 2,388(76.6) | 2,510(76.2) | 3,412(82.7) | 5,483(89.1) |  |  |
| NIH | Yes | 368(2.2) | 124(4.0) | 97(2.9) | 88(2.1) | 59(1.0) | -8.54 | <0.001 |
|  | no | 16,324(97.8) | 2,992(96.0) | 3,198(97.1) | 4,037(97.9) | 6,097(99.0) |  |  |
| US federal | Yes | 38(0.2) | 13(0.4) | 9(0.3) | 7(0.2) | 9(0.1) | -2.28 | 0.046 |
|  | no | 16,654(99.8) | 3,103(99.6) | 3,286(99.7) | 4,118(99.8) | 6,147(99.9) |  |  |
| Other | Yes | 13,387(80.2) | 2,251(72.2) | 2,404(73.0) | 3,317(80.4) | 5,415(88.0) | 18.02 | <0.001 |
|  | no | 3,305(19.8) | 865(27.8) | 891(27.0) | 808(19.6) | 741(12.0) |  |  |

^a^ Cochran-Armitage test for temporal trend of four time subsets.

eTable 6 Temporal trend for condition categories of interventional trials registered in ClinicalTrials.gov, 2008-2019

|  | **Trials,No.(%)** | | | | | **Z value** ^a^ | ***P* value** |
| --- | --- | --- | --- | --- | --- | --- | --- |
|  |  | **2008-2010**  **(n = 7,029)** | **2011-2013**  **(n = 7,486)** | **2014-2016**  **(n = 9,883)** | **2017-2019**  **(n = 11,738)** |  |  |
| **Condition** |  |  |  |  |  |  |  |
| Infectious | yes | 1,311(18.7) | 1,136(15.2) | 1,166(11.8) | 1,250(10.6) | -14.30 | <0.001 |
|  | no | 5,718(81.3) | 6,350(84.8) | 8,717(88.2) | 10,488(89.4) |  |  |
| Cancer | yes | 1,025(14.6) | 884(11.8) | 1,136(11.5) | 1,344(11.4) | -5.02 | <0.001 |
|  | no | 6,004(85.4) | 6,602(88.2) | 8,747(88.5) | 10,394(88.6) |  |  |
| Immune | yes | 1,068(15.2) | 934(12.5) | 1,064(10.8) | 1,222(10.4) | -8.69 | <0.001 |
|  | no | 5,961(84.8) | 6,552(87.5) | 8,819(89.2) | 10,516(89.6) |  |  |
| Respiratory | yes | 985(14.0) | 918(12.3) | 991(10.0) | 961(8.2) | -11.72 | <0.001 |
|  | no | 6,044(86.0) | 6,568(87.7) | 8,892(90.0) | 10,777(91.8) |  |  |
| Mental | yes | **605(8.6)** | **676(9.0)** | **971(9.8)** | **1,292(11.0)** | **5.08** | **<0.001** |
|  | no | 6,424(91.4) | 6,810(91.0) | 8,912(90.2) | 10,446(89.0) |  |  |
| Digestive | yes | 607(8.6) | 631(8.4) | 779(7.9) | 786(6.7) | -4.58 | <0.001 |
|  | no | 6,422(91.4) | 6,855(91.6) | 9,104(92.1) | 10,952(93.3) |  |  |

^a^ Cochran-Armitage test for temporal trend of four time subsets.

eTable 7 Temporal trend for condition categories of observational studies registered in ClinicalTrials.gov, 2008-2019

|  | **Trials,No.(%)** | | | | | **Z value** ^a^ | ***P*** **value** |
| --- | --- | --- | --- | --- | --- | --- | --- |
|  |  | **2008-2010**  **(n = 3,116)** | **2011-2013**  **(n = 3,295)** | **2014-2016**  **(n = 4,125)** | **2017-2019**  **(n = 6,156)** |  |  |
| **Condition** |  |  |  |  |  |  |  |
| Infectious | yes | 458(14.7) | 458(13.9) | 469(11.4) | 676(11.0) | -5.07 | <0.001 |
|  | no | 2,658(85.3) | 2,837(86.1) | 3,656(88.6) | 5,480(89.0) |  |  |
| Cancer | yes | 604(19.4) | 471(14.3) | 493(12.0) | 775(12.6) | -7.34 | <0.001 |
|  | no | 2,512(80.6) | 2,824(85.7) | 3,632(88.0) | 5,381(87.4) |  |  |
| Immune | yes | 399(12.8) | 374(11.4) | 344(8.3) | 534(8.7) | -6.04 | <0.001 |
|  | no | 2,717(87.2) | 2,921(88.6) | 3,781(91.7) | 5,622(91.3) |  |  |
| Respiratory | yes | 387(12.4) | 403(12.2) | 395(9.6) | 622(10.1) | -3.62 | <0.001 |
|  | no | 2,729(87.6) | 2,892(87.8) | 3,730(90.4) | 5,534(89.9) |  |  |
| Mental | yes | 171(5.5) | 179(5.4) | 269(6.5) | 361(5.9) | 0.96 | 0.340 |
|  | no | 2,945(94.5) | 3,116(94.6) | 3,856(93.5) | 5,795(94.1) |  |  |
| Digestive | yes | 266(8.5) | 288(8.7) | 353(8.6) | 580(9.4) | 1.25 | 0.210 |
|  | no | 2,850(91.5) | 3,007(91.3) | 3,772(91.4) | 5,576(90.6) |  |  |

^a^ Cochran-Armitage test for temporal trend of four time subsets.

eTable 8 Characteristics of reporting results within different study types, 2008-2019

|  | **Trials, No.(%)** | | | | | |
| --- | --- | --- | --- | --- | --- | --- |
|  | **All studies** | | **Observational** | | **Interventional** | |
|  | **Has Results**  **(n = 6,125)** | **No Results Available**  **(n = 46,935)** | **Has Results**  **(n = 722)** | **No Results Available**  **(n = 15,970)** | **Has Results**  **(n = 5,403)** | **No Results Available**  **(n = 30,733)** |
| **Lead sponsor** |  |  |  |  |  |  |
| Industry | 3,080(30.4) | 7,053(69.6) | 458(15.8) | 2,441(84.2) | 2,622(36.9) | 4,479(63.1) |
| NIH | 132(15.7) | 709(84.3) | 3(0.8) | 365(99.2) | 129(27.5) | 340(72.5) |
| US federal | 52(20.0) | 208(80.0) | 7(18.4) | 31(81.6) | 45(20.5) | 175(79.5) |
| Other | 2,861(6.8) | 38,965(93.2) | 254(1.9) | 13,133(98.1) | 2,607(9.2) | 25,739(90.8) |
| **Enrollment** |  |  |  |  |  |  |
| 1-100 | 3,349(12.3) | 23,883(87.7) | 218(3.3) | 6,400(96.7) | 3,131(15.2) | 17,483(84.8) |
| 101-1,000 | 2,248(12.1) | 16,259(87.9) | 290(4.5) | 6,222(95.5) | 1,958(16.3) | 10,037(83.7) |
| ＞1,000 | 528(9.5) | 5,025(90.5) | 214(6.8) | 2,922(93.2) | 314(13.0) | 2,103(87.0) |
| **Phase** |  |  |  |  |  |  |
| Early Phase 1 | 13(3.2) | 397(96.8) | - | - | 13(3.2) | 397(96.8) |
| NA | 1,554(8.1) | 17,561(91.9) | - | - | 1,554(8.1) | 17,561(91.9) |
| Phase 1 | 190(9.3) | 1,853(90.7) | - | - | 190(9.3) | 1,853(90.7) |
| Phase 1/2 | 244(17.5) | 1,149(82.5) | - | - | 244(17.5) | 1,149(82.5) |
| Phase 2 | 1,069(24.8) | 3,243(75.2) | - | - | 1,069(24.8) | 3,243(75.2) |
| Phase 2/3 | 129(14.3) | 771(85.7) | - | - | 129(14.3) | 771(85.7) |
| Phase 3 | 1,488(32.9) | 3,037(67.1) | - | - | 1,488(32.9) | 3,037(67.1) |
| Phase 4 | 716(20.8) | 2,722(79.2) | - | - | 716(20.8) | 2,722(79.2) |

eTable 9 Supplementary analysis

|  | **Trials, No.(%)** | | **$\chi^{2}$** | ***P* value** |
| --- | --- | --- | --- | --- |
|  | **Only children**  **(n = 13,364)** | **Children and Adults**  **(n = 22,772)** |  |  |
| **Status_Completed** |  |  | 129.6 | <0.001 |
| Yes | 7,653(57.3) | 11,630(51.1) |  |  |
| No | 5,711(42.7) | 11,142(48.9) |  |  |
| **Intervention_Behavioral** |  |  | 114.3 |  |
| Yes | 2,661(19.9) | 3,533(15.5) |  | <0.001 |
| No | 10,703(80.1) | 19,239(84.5) |  |  |

**$\chi^{2}$** test for effect of age group influencing proportion of trials.

eTable 10 Supplementary analysis

|  | **Trials, No.(%)** | | | | | |
| --- | --- | --- | --- | --- | --- | --- |
|  | **Infectious**  **(n = 4,863)** | **Cancer**  **(n = 4,389)** | **Immune**  **(n = 4,288)** | **Respiratory**  **(n = 3,855)** | **Mental**  **(n = 3,544)** | **Mental(child)(n = 1,744)** |
| **Intervention** |  |  |  |  |  |  |
| Drug | 2,120(43.6) | 2,388(54.4) | 2,214(51.6) | 1,684(43.7) | **962(27.1)** | **568(32.6)** |
| Behavioral | 378(7.8) | 312(7.1) | 424(9.9) | 265(6.9) | **1,685(47.5)** | **806(46.2)** |

eTable 11 Supplementary analysis

|  | **Trials, No.(%)** | | **$\chi^{2}$** | ***P* value** |
| --- | --- | --- | --- | --- |
|  | **Intervention_Behavioral** | |  |  |
|  | **Yes** | **No** |  |  |
| **Child and Adult** |  |  | 3,696.1 | <0.001 |
| Mental | 1,685(47.5) | 1,859(52.5) |  |  |
| Other four conditions ^a^ | 1,379(7.9) | 16,016(92.1) |  |  |
| **Mental** |  |  | 0.8 | 0.378 |
| Only child | 806(46.2) | 938(53.8) |  |  |
| Child and Adult | 1,685(47.5) | 1,859(52.5) |  |  |

^a^ Includes infectious, cancer, immnue, respiratory.
